# Clinical Outcomes of Tanezumab With Different Dosages for Patient With Osteoarthritis: Network Meta-Analysis

**DOI:** 10.3389/fphar.2021.614753

**Published:** 2021-06-11

**Authors:** Rui Hu, Ya-Feng Song, Zhi-Yan Yang, Chao Zhang, Bo Tan

**Affiliations:** ^1^ Department of Orthopedics, Taihe Hospital, Hubei University of Medicine, Shiyan, China; ^2^ Department of Personnel Office, Taihe Hospital, Hubei University of Medicine, Shiyan, China; ^3^ Center for Evidence-Based Medicine and Clinical Research, Taihe Hospital, Hubei University of Medicine, Shiyan, China; ^4^ Department of Orthopedics, Sichuan Provincial People’s Hospital, University of Electronic Science and Technology of China, Chengdu, China

**Keywords:** osteoarthritis, tanezumab, WOMAC, clinical outcomes, systematic review

## Abstract

**Background:** Osteoarthritis (OA) high disability rate will increase as people getting older, and is the most prevalent form of arthritis in the future. This study identified the clinical effects of optimum doses of tanezumab for patients with OA.

**Method:** Three electronic databases were searched up until January 15, 2021. The mean difference (MD) or odds ratio (OR) was considered an effect measure. The design-by-treatment interaction model was adopted for network meta-analyses. Analyses were conducted using WinBUGS 1.4.3 and R 4.0.5 software.

**Results:** nine publications with 10 studies were included. Compared with placebo in network meta-analysis, except the outcomes of Western Ontario and McMaster Universities Osteoarthritis (WOMAC) stiffness subscale and joints replaced, all dosages of tanezumab in the other effectiveness outcome were superior to placebo, and the difference was statistically significant. However, there was no statistical difference among all different doses of tanezumab. Compared with placebo, except the outcomes of adverse events (AEs) and AEs of abnormal peripheral sensation, all different dosages of tanezumab weren’t superior to placebo in the other effectiveness outcome, and the difference was statistically significant. The 10 mg of tanezumab with highest SUCRA had the best effect, but it was associated with a higher safety event. Compared with placebo, except the outcomes of WOMAC stiffness subscale and joints replaced, all dosages of tanezumab in the other effectiveness outcome were superior to placebo, and the difference was statistically significant. Compared with placebo, except for the outcomes of AEs and AEs of abnormal peripheral sensation, all dosages of tanezumab in the other effectiveness outcome were superior to placebo, and the difference was statistically significant. Other direct comparisons showed no statistical difference.

**Conclusion:** This study recommended that clinicians should give priority to the treatment of OA patients with a low dose of 2.5 mg according to the patient’s condition and actual situation. If the effect using tanezumab with 2.5 mg is not satisfactory, the increase up to 10 mg should be carefully pondered, because of a more unbalanced risk/benefit ratio.

## Introduction

Osteoarthritis (OA) is unpredictable chronic joint disease, which usually lies dormant for a long time (Ekman et al., 2014; Karsdal et al., 2019; Berenbaum et al., 2020). Patients with OA will increase as people getting older. Once elderly OA patients combined with diabetes, these are more prone to experience adverse effects (Ekman et al., 2014; Kan et al., 2016; Tive et al., 2019).

At present, drug therapy is still the most important intervention method and challenge (Kan et al., 2016). Non-steroidal anti-inflammatory drugs (NSAIDs) and opioids are representative drugs improving and relieving the pain of OA, but they may increase adverse events (AEs). According to relevant studies, based on the mechanism that anti NGF may reduce OA related pain, nerve growth factor (NGF) antagonists may become candidate drugs (Chevalier et al., 2013). Subsequently, related studies (Brown et al., 2012; Brown et al., 2013; Schnitzer et al., 2015; Berenbaum et al., 2020) reported the clinical efficacy and safety of tanezumab. Based on some studies (Karsdal et al., 2019; Schnitzer et al., 2019; Tive et al., 2019; Patel et al., 2020), transient cutaneous paresthesia was reported in some patients, while subsequent studies minimized the risk and provided a comprehensive summary of joint and nervous system AEs. Tanezumab has high affinity and specificity for NGF, which is an important carrier to transmit pain signals (Ekman et al., 2014). In adults, reducing NGF sensitivity results in decreased peripheral receptor sensitivity and decreased neuropeptide levels (Tive et al., 2019). Related evidences suggested that NGF injection into the skin can cause pain (Ekman et al., 2014; Schnitzer and Marks, 2015; Tive et al., 2019). Based on the results of the latest randomized controlled trials (RCT) from Berenbaum’s phase III study in 2020 (Berenbaum et al., 2020), the tanezumab 5 mg statistically significantly improved pain, physical function and Patient’s Global Assessment of OA, while tanezumab 2.5 mg only achieved two co-primary end points. However, rapidly progressive osteoarthritis occurred more frequently with tanezumab 5 mg than tanezumab 2.5 mg. Moreover, the results of stratified meta-analysis in 2020 (Yu et al., 2020) showed that there was no difference in benefit between 2.5 and 5 mg of tanezumab, except for the outcome of rapidly progressive osteoarthritis. In addition, no complementary analysis of different doses was performed in other meta-analyses about tanezumab for OA (Kan et al., 2016; Chen et al., 2017; Fan et al., 2020), leading to a lack of evidence of a dose-response relationship to guide clinical application. A conclusion of RCT from Nagashima (Nagashima et al., (2011)) showed that at doses of 10 and 50 mg/kg, the effect of tanezumab on these efficacy endpoints was not substantially different from placebo. A previous study in patients with knee OA from the United States (Lane et al., 2010) showed tanezumab to be effective in reducing pain at doses of 10 and 50 mg/kg. The reason for the lack of efficacy of tanezumab at these doses in this study is unclear. By contrast, we observed a generally dose-related incidence of AEs of abnormal peripheral sensation (Lane et al., 2010; Brown et al., 2012). To demonstrate the clinical effects of optimum dosages of tanezumab for patients with OA, this research investigated the clinical efficacy and safety of tanezumab of clinical outcomes to guide clinicians to make the best decisions based on network meta-analysis.

## Methods

### Search Strategy

Three electronic academic databases, including the Medline and EMbase from Ovid, the Cochrane Library, and Web of Science were searched up until January 15, 2021, using “Osteoarthritis,” “Osteoarthrosis,” “Arthritis, Degenerative,” “Degenerative Arthritides,” “Arthrosis,” “Osteoarthrosis Deformans,” and “Tanezumab”.

### Inclusion Criteria

The following inclusion criteria for clinical trials were adopted: 1) Adult patients with OA of knee or hip; 2) The intervention group was treated with tanezumab, which must be fixed dosage in order to avoid differences or inconsistences in dose changes; 3) The control group was placebo; 4) Effective outcomes, including pain subscale, physical function subscale, stiffness subscale and pain reduction (≥30, ≥50, ≥70, and ≥90%) based on the Western Ontario and McMaster Universities Osteoarthritis (WOMAC), patient’s global assessment of OA, and joints replaced; Safety outcomes, including AEs, serious AEs, discontinued due to AEs, treatment-related AEs, AEs of abnormal peripheral sensation, and new or worsened abnormalities. 4) The included study must be RCT.

Studies were excluded based on the following criteria: 1) Duplicate studies 2) The data could not be extracted or obtained through contact with the author; 3) Studies with insufficient data for statistical analysis; 4) Studies without available full text.

### Data Extraction and Methodological Quality

The study design included: patient characteristics, interventions, controls, and outcomes. The data acquisition was done independently by two authors. The methodological quality was assessed using the Cochrane Collaboration’s tool (Higgins and Green, 2011).

### Statistical Analysis

The weighted mean difference (MD) was considered an effect size for continuous outcomes (Higgins and Green, 2011), and odds ratio (OR) was employed for other dichotomous outcomes (Higgins and Green, 2011). The statistical test for heterogeneity was performed, and I^2^ > 40% and *p* < 0.1 were considered as heterogeneity as well. For outcomes with high heterogeneity, meta-regression analysis was used to explore confounding factors in order to identify potential sources of heterogeneity (Higgins and Green, 2011).

The design-by-treatment interaction model (Günhan et al., 2018) was adopted for network meta-analyses. By using non-informative priors with vague normal (mean 0, variance 10,000) and uniform (0–5) prior distributions for parameters such as the means and standard deviations (Lu and Ades, 2004). First, 10,000 simulations were performed, and then we generated an additional 60,000 simulations with three sets of different initial values and sheared the first 10,000 simulations as the burn-in period in our model. We used the Brooks-Gelman-Rubin statistical method for assessing model convergence. Based on 50,000 simulations with 10 thin, the point estimate adopted the median of the posterior distribution, and the corresponding 95% credible interval (CrIs) used the 2.5th and 97.5th percentiles of the posterior distributions, which were interpreted in a similar fashion as conventional 95% confidence intervals.

We assessed loop inconsistency in the network meta-analysis (Salanti, 2012). To summarize probabilities, we used the surface under the cumulative ranking curve (SUCRA) to provide a summary statistic for the cumulative ranking (Salanti et al., 2011). All data analyses were conducted using WinBUGS 1.4.3 and R 4.0.5 software. The latest Preferred Reporting Items for Systematic Reviews and Meta-Analyses (PRISMA) extension statement (Hutton et al., 2015) for the reporting of systematic reviews and network meta-analysis was used.

## Results

### Characteristics and Methodological Quality of Included Studies

**FIGURE 1:**
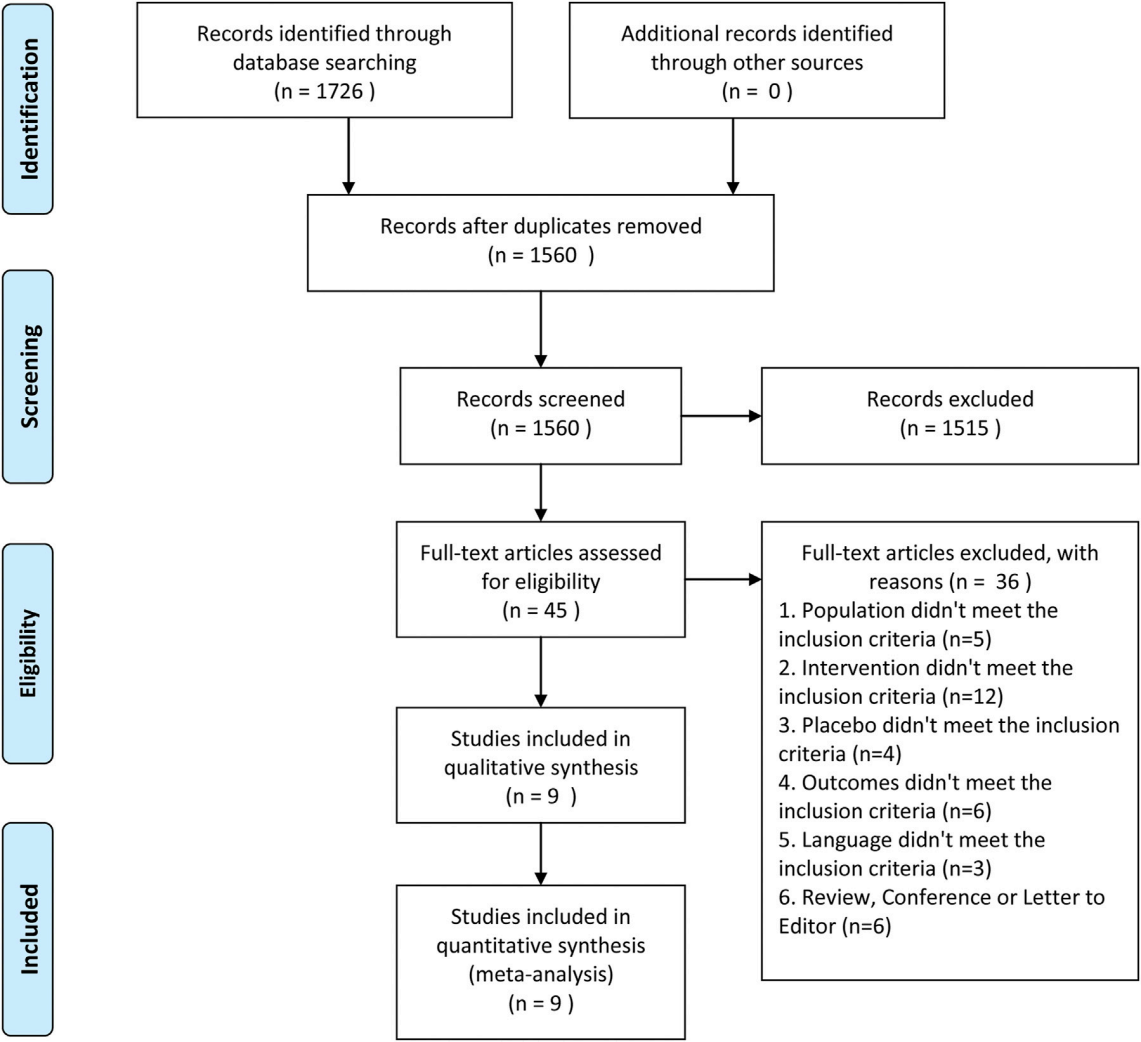
Outline of screening and identification of studies.

1,726 individual studies were searched. After inclusion and exclusion criteria, 45 full texts were assessed for eligibility. Finally, nine RCTs (Brown et al., 2012; Brown et al., 2013; Spierings et al., 2013; Balanescu et al., 2014; Brown et al., 2014; Ekman et al., 2014; Schnitzer et al., 2015; Schnitzer et al., 2019; Berenbaum et al., 2020) with 10 studies involving 7,004 patients were involved in the meta-analysis (Figure 1). Table 1 displayed the essential characteristic of included 10 studies. The result of methodological quality is shown in Table 2.

**TABLE 1:** Essential characteristic of included studies.

Study	Year	Country	Age	Sample	Kellgren-Lawrence grade	Baseline Patient’s global assessment of OA	Inflamed joint	WOMAC pain subscale score	WOMAC physical function subscale score	Duration of joint disease	Tanezumab	Placebo	Follow-up (Week)
Spierings	2013	United States	57.0(29–74)/57.8(28–75)/57.2(28–75)	150/161/141	1: 0/0/0; 2: 73/78/67; 3: 55/60/56; 4: 22/23/18	Good: 0/0/0; Fair: 81/81/72; Poor: 53/66/54; Very poor: 16/14/15	Knee: 117/116/117; Hip: 33/45/24	7.64 ± 1.31/7.87 ± 1.26/7.75 ± 1.20	7.05 ± 1.54/7.32 ± 1.59/7.17 ± 1.51	7.5(0–34)/7.6(0–40)/7.4(0–43)	10 mg; 5 mg 8-week interval for 16 weeks	Placebo	8
Schnitzer	2019	United States	61.2(32–83)/60.9(27–84)/60.4(31–85)	233/231/232	1: 0/1/0; 2: 59/60/65; 3: 105/101/98; 4: 68/69/69	Good: 0/1/0; Fair: 125/144/134; Poor: 92/74/89; Very poor: 16/12/9	Knee: 198/197/199; Hip: 35/34/33	7.3(5.0–10.0)/7.1(4.8–10.0)/7.3(4.2–10.0)	7.4(3.2–9.9)/7.2(5.1–9.9)/7.4(4.4–10.0)	NR	2.5 mg administered at baseline and 5 mg at week 8; 2.5 mg administered at baseline and week 8	Placebo	16, 24
Berenbaum	2020	Europe and Japan	65.2 ± 10.2/65.2 ± 8.4/64.2 ± 9.6	284/283/282	1: 0/0/0; 2: 58/49/59; 3: 121/131/123; 4: 105/101/100	Very good: 1/0/0; Good: 1/0/0; Fair: 136/132/145; Poor: 129/129/117; Very poor: 17/21/19	Knee: 705; Hip: 144	6.6 ± 0.89/6.7 ± 0.94/6.59 ± 0.94	6.76 ± 0.88/6.77 ± 0.87/6.67 ± 0.87	6.7/6.0/7.4	5 mg, 2.5 mg subcutaneously at baseline, week 8 and week 16	Placebo	24
Balanescu	2014	Austria, France, Germany, Poland, Romania, Russia, Spain, Sweden, Ukraine and the United Kingdom	63.1(43–85)/62.2(41–80)/62.1(35–84)/62.3(39–86)	145/150/157/152	1: 0/0/0/0; 2: 73/64/77/68; 3: 62/71/68/68; 4: 10/15/12/16	3.37 ± 0.55/3.43 ± 0.56/3.28 ± 0.46/3.39 ± 0.57	Knee: 117/115/121/121; Hip: 28/35/36/31	5.87 ± 1.30/5.76 ± 1.32/5.78 ± 1.36/6.06 ± 1.23	5.91 ± 1.36/5.90 ± 1.22/5.86 ± 1.51/6.24 ± 1.45	6.6(0.1–30.0)/6.7(0.2–29.9)/6.1(0.0–40.1)/6.1(0.1–29.8)	10 mg, 5 mg, 2.5 mg intravenous infusion every 8 weeks for a total of three doses	Placebo	32
Brown	2012	United States	61.4(38–87)/62.1(32–85)/60.8(34–84)/62.2(39–84)	174/172//172/172	1: 0/0/0/0; 2: 71/64/64/68; 3: 77/89/74/82; 4: 26/18//31/22	3.5/3.5/3.5/3.4	Knee: 690	7.0/7.2/7.2/7.1	6.7/6.9/6.9/6.6	9.5(0–49)/7.5(0–35)/7.3(0–37)/8.2(0–39)	10 mg, 5 mg, 2.5 mg intravenous infusion at 8-week intervals on 3 occasions during the study	Placebo	24
Brown	2013	United States	63.3(35–92)/61.8(21–88)/62.4(26–88)/61.9(31–88)	157/154/155/155	1: 0/1/0/0; 2: 67/72/71/73; 3: 58/54/53/56; 4: 32/27/31/26	3.5/3.5/3.6/3.5	Hip: 621	7.3/7.2/7.2/7.3	6.8/6.8/6.8/6.8	5.6(0–59)/6.3(0–40)/6.0(0–59)/5.6(0–50)	10 mg, 5 mg, 2.5 mg intravenous infusion at 8-week intervals on 3 occasions during the study	Placebo	24
Brown	2014	United States	58.0 ± 9.0/57.8 ± 8.3/56.3 ± 10.2	74/73/72	1: 1/0/1; 2: 22/25/31; 3: 26/18/20; 4: 10/11/8; miss: 15/19/12	Good: 0/0/1; Fair: 53/54/46; Poor: 21/17/23; Very poor: 0/2/2	Knee: 60/62/63; Hip: 14/11/9	6.45 ± 1.34/5.99 ± 1.49/6.54 ± 1.55	6.19 ± 1.62/6.04 ± 1.55/6.52 ± 1.70	NR	Intravenous injections of tanezumab 5 mg and tanezumab 10 mg, administered once every 8 weeks over 24 weeks (three injections)	Placebo	24
Ekman-1015	2014	United States	61.1 ± 10.3/61.1 ± 10.1/60.9 ± 10.1	208/206/208	1: 0/0/0; 2: 98/76/89; 3: 90/108/91; 4: 20/22/28	NR	Knee: 208/206/208; Hip: 0/0/0	7.23 ± 1.40/7.29 ± 1.46/7.20 ± 1.40	6.82 ± 1.50/6.84 ± 1.71/6.82 ± 1.54	NR	10 and 5 mg administered on day 1 and day 57 (Week 8)	Placebo	16
Ekman-1018	2014	United States	59.2 ± 10.3/59.8 ± 9.6/60.1 ± 9.4	209/211/209	1: 0/0/0; 2: 101/104/107; 3: 72/77/79; 4: 36/30/22	NR	Knee: 168/168/168; Hip: 41/43/41	7.37 ± 1.39/7.27 ± 1.38/7.41 ± 1.38	7.09 ± 1.52/6.83 ± 1.56/7.04 ± 1.49	NR	10 and 5 mg administered on day 1 and day 57 (Week 8)	Placebo	16
Schnitzer	2015	United States	62.0 ± 10.2/61.9 ± 9.7/61.3 ± 9.3	542/541/539	1: 0/0/0; 2: 187/181/197; 3: 203/191/217; 4: 152/169/125	Naproxen: 3.4/3.4/3.4; Celecoxib: 3.5/3.4/3.4	Knee: 449/449/446; Hip: 93/92/93	Naproxen: 6.5/6.4/6.3; Celecoxib: 6.4/6.5/6.3	Naproxen: 6.5/6.5/6.3; Celecoxib: 6.6/6.7/6.5	7.1(0–49.7)/7.3(0–43.8)/7.5(0–49.7)	10 and 5 mg every 8 weeks to a maximum of seven administrations	Placebo	16

Note: OA, Osteoarthritis; PGA, Patient’s Global Assessment; WOMAC, Western Ontario and McMaster Universities Osteoarthritis.

**TABLE 2:** Risk of bias for included studies.

Trial or author	Year	Random sequence generation	Allocation concealment	Blinding of participants and personnel	Blinding of outcome assessment	Incomplete outcome data	Selective reporting	Other bias
Spierings	2013	Low risk	Low risk	Low risk	Low risk	Low risk	Low risk	Unclear
Schnitzer	2019	Low risk	Low risk	Low risk	Low risk	Low risk	Low risk	Unclear
Berenbaum	2020	Low risk	Low risk	Unclear	Low risk	Low risk	Low risk	Unclear
Balanescu	2014	Low risk	Low risk	Low risk	Low risk	Low risk	Low risk	Unclear
Brown	2012	Low risk	Low risk	Low risk	Low risk	Low risk	Low risk	Unclear
Brown	2013	Low risk	Low risk	Low risk	Low risk	Low risk	Low risk	Unclear
Brown	2014	Low risk	Low risk	Unclear	Low risk	Low risk	Low risk	Unclear
Ekman-1015	2014	Low risk	Low risk	Low risk	Low risk	Low risk	Low risk	Unclear
Ekman-1018	2014	Low risk	Low risk	Low risk	Low risk	Low risk	Low risk	Unclear
Schnitzer	2015	Low risk	Low risk	Low risk	Low risk	Low risk	Low risk	Unclear

### Network Meta-Analysis

#### Efficiency Outcomes

**FIGURE 2:**
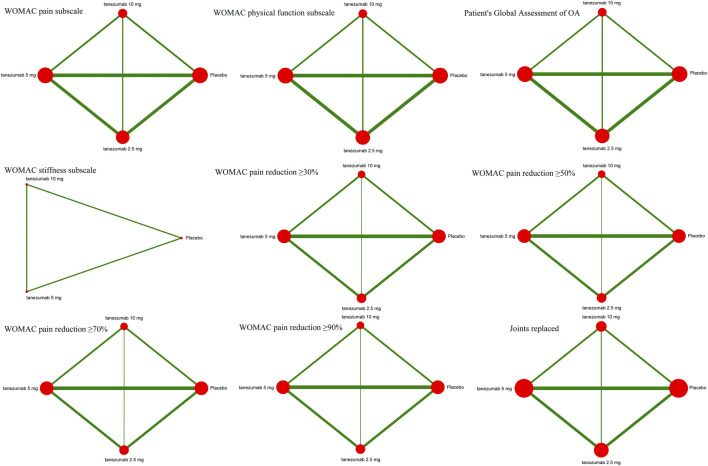
The network of eligible studies in efficiency outcomes. The node sizes correspond to the sample size that investigated the treatments. Directly comparable treatments are linked with a line, and the thickness of the line corresponds to the sum of the sample size in each pairwise treatment comparison.

Figure 2 indicates the network of eligible studies with different dosages of tanezumab and placebo from all efficiency outcomes. Supplementary Figure S1 shows the results of loop consistency for all efficiency outcomes, showing no inconsistency. Compared with placebo, except the outcomes of WOMAC stiffness subscale and joints replaced, all dosages of tanezumab in the other effectiveness outcome were superior to placebo, and the difference weren’t statistically significant (Table 3). In particular, tanezumab with 10 mg (MD = −0.83, 95%CrIs = −1.04, −0.61), 5 mg (MD = −0.81, 95%CrIs = −1.01, −0.61), and 2.5 mg (MD = −0.68, 95%CrIs = −0.93, −0.41) were superior to placebo for WOMAC pain subscale. In WOMAC physical function subscale, tanezumab with 10 mg (MD = −0.93, 95%CrIs = −1.12, −0.74), 5 mg (MD = −0.92, 95%CrIs = −1.10, −0.73), and 2.5 mg (MD = −0.69, 95%CrIs = −0.93, −0.44) were superior to placebo. In patient’s global assessment of OA, tanezumab with 10 mg (MD = −0.25, 95%CrIs = −0.33, −0.17), 5 mg (MD = −0.25, 95%CrIs = −0.33, −0.18), and 2.5 mg (MD = −0.20, 95%CrIs = −0.30, −0.10) were superior to placebo. In WOMAC pain reduction ≥30%, tanezumab with 10 mg (OR = 1.57, 95%CrIs = 1.26, 1.95), 5 mg (OR = 1.58, 95%CrIs = 1.31, 1.90), and 2.5 mg (OR = 1.37, 95%CrIs = 1.07, 1.74) were superior to placebo. In WOMAC pain reduction ≥50%, tanezumab with 10 mg (OR = 1.90, 95%CrIs = 1.51, 2.39), 5 mg (OR = 1.73, 95%CrIs = 1.42, 2.09), and 2.5 mg (OR = 1.54, 95%CrIs = 1.21, 1.96) were superior to placebo. In WOMAC pain reduction ≥70%, tanezumab with 10 mg (OR = 1.96, 95%CrIs = 1.47, 2.64), 5 mg (OR = 1.85, 95%CrIs = 1.47, 2.37), and 2.5 mg (OR = 1.65, 95%CrIs = 1.22, 2.22) were superior to placebo. In WOMAC pain reduction ≥90%, tanezumab with 10 mg (OR = 1.98, 95%CrIs = 1.24, 3.26), 5 mg (OR = 2.35, 95%CrIs = 1.60, 3.56), and 2.5 mg (OR = 1.97, 95%CrIs = 1.21, 3.24) were superior to placebo. There was no statistical difference among all doses of tanezumab.

**TABLE 3:** Results of network meta-analysis and direct comparison meta-analysis for efficiency outcomes.

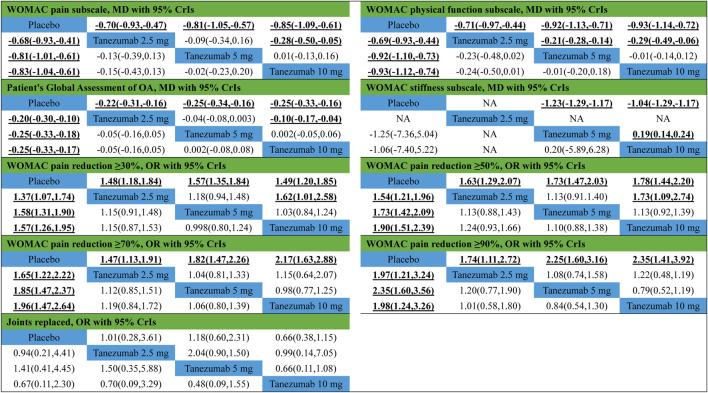

Note: Bold values indicate the significance results. Comparisons between treatments should be read from left to right and the estimate is in the cell in common between the upper-left-defining treatment and the lower-right-defining treatment. The results in the lower left corner refer to network meta-analysis, and the results in the upper right corner refer to direct comparison meta-analysis. WOMAC, the Western Ontario and McMaster Universities Osteoarthritis; OA, Osteoarthritis; NA, Not Applicable.

#### Safety Outcomes

**FIGURE 3:**
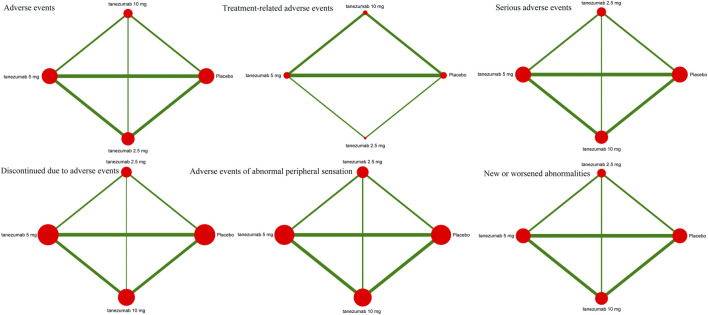
The network of eligible studies in safety outcomes. The node sizes correspond to the sample size that investigated the treatments. Directly comparable treatments are linked with a line, and the thickness of the line corresponds to the sum of the sample size in each pairwise treatment comparison.

Figure 3 indicates the network of eligible studies with different dosages of tanezumab and placebo from all safety outcomes. Compared with placebo, except the outcomes of serious AEs, treatment-related AEs and new or worsened abnormalities, all the active drugs of tanezumab in the other effectiveness outcomes weren’t superior to placebo, and it were statistically significant in other outcomes (Table 4). In outcomes of AEs, the incidence of tanezumab with 10 mg (OR = 1.41, 95%CrIs = 1.22, 1.64), tanezumab with 5 mg (OR = 1.27, 95%CrIs = 1.11, 1.45), and tanezumab with 2.5 mg (OR = 1.44, 95%CrIs = 1.20, 1.73) was significantly higher than with placebo. In outcomes of AEs of abnormal peripheral sensation, the incidence of tanezumab with 10 mg (OR = 3.97, 95%CrIs = 2.34, 6.86), tanezumab with 5 mg (OR = 2.71, 95%CrIs = 1.58, 4.66), and 2.5 mg (OR = 3.23, 95%CrIs = 1.67, 6.37) was significantly higher than with placebo. In discontinued due to AEs, compared with the other groups, tanezumab with 10 mg (OR = 2.01, 95%CrIs = 1.41, 3.03) had a higher discontinuation rate. There was no statistical difference among all doses of tanezumab.

**TABLE 4:** Results of network meta-analysis and direct comparison meta-analysis for safety outcomes.

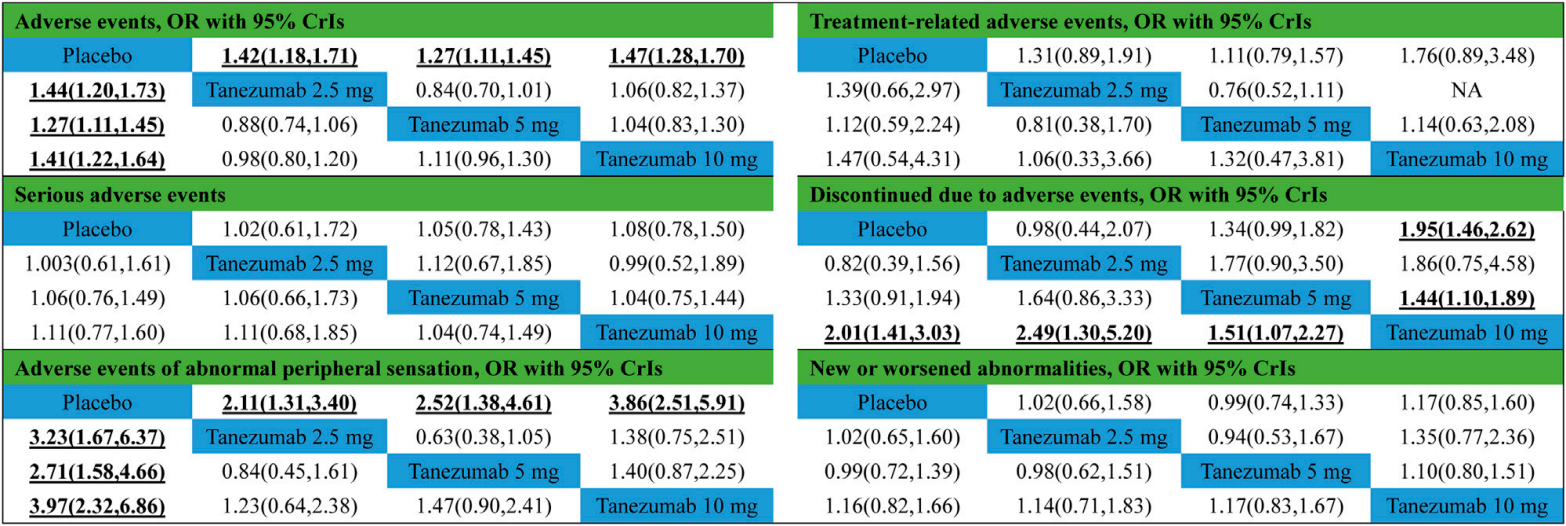

Note: Bold values indicate the significance results. Comparisons between treatments should be read from left to right and the estimate is in the cell in common between the upper-left-defining treatment and the lower-right-defining treatment. The results in the lower left corner refer to network meta-analysis, and the results in the upper right corner refer to direct comparison meta-analysis. NA, Not Applicable.

### Direct Comparison meta-Analysis

#### Efficiency Outcomes

Compared with placebo, except for the outcomes of WOMAC stiffness subscale and joints replaced, all dosages of tanezumab in the other effectiveness outcome were superior to placebo, and the difference was statistically significant (Table 3). Compared with tanezumab with 2.5 mg, tanezumab with 10 mg were more effective in outcomes of WOMAC pain subscale (MD = −0.28, 95%CrIs = −0.50, −0.05), WOMAC physical function subscale (MD = −0.29, 95%CrIs = −0.49, −0.06), patient’s global assessment of OA (MD = −0.10, 95%CrIs = −0.17, −0.04), WOMAC pain reduction ≥30% (OR = 1.62, 95%CrIs = 1.01, 2.58), and WOMAC pain reduction ≥50% (OR = 1.73, 95%CrIs = 1.09, 2.74). However, the tanezumab with 5 mg (MD = 0.19, 95%CrIs = 0.14, 0.24) was superior to tanezumab with 10 mg in WOMAC stiffness subscale. Other direct comparisons showed no statistical difference.

#### Safety Outcomes

Compared with placebo, except for the outcomes of AEs and AEs of abnormal peripheral sensation, all dosages of tanezumab in the other effectiveness outcome were superior to placebo, and the difference was statistically significant (Table 4). Compared with tanezumab with 10 mg, tanezumab with 5 mg (OR = 1.44, 95%CrIs = 1.10, 1.89) and placebo (OR = 1.95, 95%CrIs = 1.46, 2.62) have high incidences in outcomes for discontinuation based on AEs. However, other direct comparisons showed no statistical difference.

### Rank Probabilities

**FIGURE 4:**
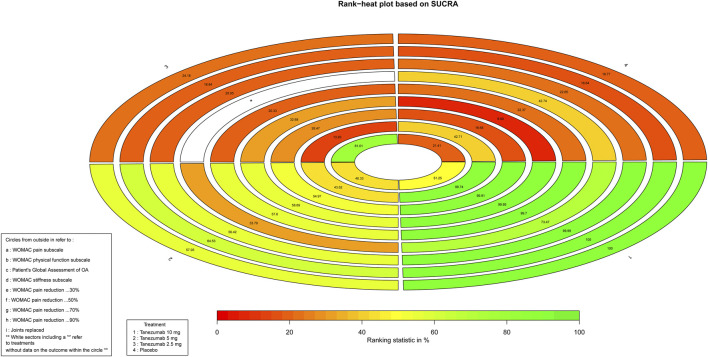
The Rank-heat plot of SUCRA for efficiency outcomes. Each sector is colored according to the SUCRA value of the corresponding treatment and outcome. The scale consists of the transformation of three colors red (0%), yellow (50%), and green (100%), and each color is associated with a different pattern. Uncolored sectors show that the underlying treatment was not included in the network meta-analysis for the particular outcome. SUCRA: Surface under the cumulative ranking.

As for the efficiency outcomes, Figure 4 indicates the ranking of tanezumab with the three doses under study. With the exception of joints replacement, all other efficacy outcomes showed that the 10 mg dose of tanezumab with highest SUCRA had the best effect, and the placebo with lowest SUCRA had the worst.

**FIGURE 5:**
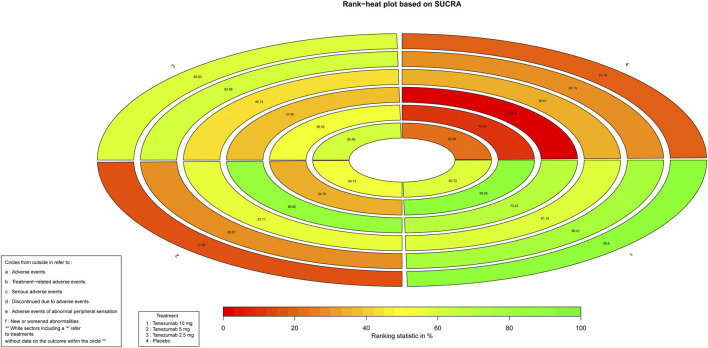
The Rank-heat plot of SUCRA for safety outcomes. Each sector is colored according to the SUCRA value of the corresponding treatment and outcome. The scale consists of the transformation of three colors red (0%), yellow (50%), and green (100%), and each color is associated with a different pattern. Uncolored sectors show that the underlying treatment was not included in the network meta-analysis for the particular outcome. SUCRA: Surface under the cumulative ranking.

As for safety outcomes, Figure 5 indicates the ranking of tanezumab with 10 mg, tanezumab with the three doses under study. With the exception of new or worsened abnormalities, all other safety outcomes showed that the 10 mg dose of tanezumab with highest SUCRA was associated with a higher safety event.

### Heterogeneity and Inconsistency Assessment

Analysis of the heterogeneity in all direct comparisons revealed that significant heterogeneity was mainly distributed in efficiency outcomes, including WOMAC pain subscale, WOMAC physical function subscale, and patient’s global assessment of OA. Regression meta-analysis of variables, including age, sample, percentage of different Kellgren-Lawrence grade, average value of WOMAC Pain subscale score, average value of WOMAC Physical Function subscale score, and duration of joint disease, showed no significant difference (*p* > 0.05) in outcomes with high heterogeneity. It’s worth noting that, for the inconsistency assessment in network meta-analysis, Supplementary Figures S1, S2 reprot the results of loop consistency for all efficiency and safety outcomes, showing no inconsistency.

## Discussion

NGF is a neurotrophic factor involved in pain signal transduction and gene expression of nociceptors (Zhu and Oxford, 2007). NGF has been shown to contribute to the clinical symptoms of pain hypersensitivity, which is commonly seen in inflammatory and chronic pain states (Ghilardi et al., 2012). NGF is expressed in subchondral bone in patients with OA, and is consistent with the role of symptomatic OA pain (Walsh et al., 2010). Tanezumab inhibs the binding of NGF to its receptors and is studied to treat chronic pain, such as OA and chronic low back pain (Bélanger et al., 2018). Since some patients in previous tanezumab studies reported transient cutaneous paresthesia, subsequent studies implemented an overall risk minimization strategy and evaluated joint and nervous system AEs in a comprehensive way (International Conference, 1997; Hochberg et al., 2016).

The present meta-analysis evaluated the clinical outcomes of tanezumab for OA. As for WOMAC indicators, our study showed that tanezumab with different dosages significantly reduced the pain, stiffness subscale, physical punction, and pain reduction of WOMAC and patient’s global assessment of OA. Although our meta-analysis did not directly compare tanezumab with other active pharmaceuticals, other studies (Spierings et al., 2013) showed that compared with NSAIDs or oxycodone, tanezumab treatment showed higher efficacy rates. Joint replacement is the treatment for joint failure. Notably, the present study showes that different doses of tanezumab do not increase joint replacement.

In line with a previous meta analysis (Yu et al., 2020), our study also found that different doses of tanezumab did not increase joint replacement. This also confirmed that the treatment with tanezumab does not increase the risk of osteonecrosis. However, a systemic review and meta-analysis of randomized phase III clinical trials (Yu et al., 2020) showed that tanezumab had a higher rate of rapidly progressive OA (RPOA) than the NSAIDs and opioids group, and 10 mg tanezumab combined with NSAIDs had the highest estimated rate of RPOA, which is also a contributor to joint replacement. It has been suggested that the reason why tanezumab causes RPOA may be that pain relief promotes an increase in joint motion, which may inadvertently lead to joint overload (Dimitroulas et al., 2017; Watt and Gulati, 2017). In conclusion, the mechanism is still unknown, and more studies are needed to pay attention to this, so as to provide reference for clinical use.

At the same time, we also found that the higher the dose, the more significant the target efficacy of WOMAC. This conclusion has also been reported by other studies (Brown et al., 2014; Ekman et al., 2014; Schnitzer and Marks, 2015). However, no differences in the benefits were found in the benefits of all drug doses, including 2.5, 5, and 10 mg. This also makes us to believe that the high dose investment does not bring a high profit return. In the previous meta-analyses, except the study published by Chen in 2016 (Chen et al., 2017), which recommended 2.5 mg as the optimal dose, the other three meta-analyses (Kan et al., 2016; Fan et al., 2020; Yu et al., 2020) did not provide the conclusion of the optimal dose.

On the contrary, other researches (Spierings et al., 2013; Balanescu et al., 2014) reported that tanezumab had certain AEs and leads to an increase in the withdrawal rate associated with AEs. In our results, tanezumab had a higher proportion of AEs than placebo. However, there was no statistical difference in the incidence of adverse events between different doses. As for treatment-related AEs and serious AEs, no obvious significant increase was found in tanezumab with all dosages, but the tanezumab with 10 mg significantly increased treatment discontinuation due to AEs. This may be due to the fact that the number of studies and sample sizes included in the outcome of treatment-related adverse events were too small to meet expectations for the statistical power of the current results. Of concern, tanezumab, has high rates of AEs of abnormal peripheral sensation, but did not increase other rate of new or worsened abnormalities.

Heterogeneity tests based on 10 RCT studies showed that the dominant heterogeneity was in the effectiveness outcome, including WOMAC pain subscale, WOMAC physical function subscale, and patient’s global assessment of OA. Although relevant confounders may exist and affect the accuracy of the results, no significant confounders were found in this study based on the regression analysis. The confounding factors cannot be strictly broken down and can only be analyzed “symbolically” in the form of percentages, such as Kellgren-Lawrence grade, or the average value of WOMAC pain subscale score, which may also affect the results of regression. However, no inconsistency was found in the loop-based inconsistency detection network meta-analysis, which further ensures the reliability of results meta-analysis.

The biggest advantage of network meta-analysis is that it can comprehensively rank the effectiveness and safety of all current interventions, so as to provide a basis for clinicians to make better decisions. In order to better use relevant evidence to patients with OA, it has been a focus to minimize the occurrence of safety events while ensuring maximum effectiveness. Based on the analysis of the ranking results of this study, tanezumab with 10 mg was ranked first in both effectiveness and AE outcomes. This also brings about some contradictions and conflicts in clinical decision-making. Therefore, this study suggested that clinicians should give priority to the treatment of OA patients with a low dose of 2.5 mg according to the patient’s condition and actual situation. If the effect using tanezumab with 2.5 mg is not satisfactory, its dose can be increased to 5 or 10 mg, but the relevant safety events must be monitored more intensively.

The clinical outcomes of tanezumab in the management of OA were comprehensively evaluated while including as many qualified studies and sample sizes as possible in this study. This study also has some limitations. First, there are too few studies on different dosages of drugs in all outcomes, which may lead to unstable results. Secondly, unlawfully controlled confounding factors were mixed into this study, resulting in greater direct heterogeneity. However, the results of network meta-analysis prompted the existence of consistencies.

Overall, this study confirmed that tanezumab with 10 mg has a powerful effect on the treatment of OA. However, it also increases the risk of AE. Therefore, we recommend that clinicians should give priority to the treatment of OA patients with a low dose of 2.5 mg according to the patient’s condition and actual situation. If the effect using tanezumab with 2.5 mg is not satisfactory, the increase up to 10 mg should be carefully pondered, because of a more unbalanced risk/benefit ratio.

## Data Availability

The original contributions presented in the study are included in the article/Supplementary Material, further inquiries can be directed to the corresponding author.
