# Supplementary material for: Clinical Outcomes of Tanezumab With Different Dosages for Patient With Osteoarthritis: Network Meta-Analysis
Source: Front Pharmacol. 2021 Jun 11;12:614753. doi: 10.3389/fphar.2021.614753 (PMC8232525; doi:10.3389/fphar.2021.614753)

**Legends of Supplementary Figure**

**Supplementary Figure 1** The results of loop consistency for all efficiency outcomes

**Supplementary Figure 2** The results of loop consistency for all safety outcomes

**Supplementary Figure 1** The results of loop consistency for all efficiency outcomes

**WOMAC pain subscale**

**
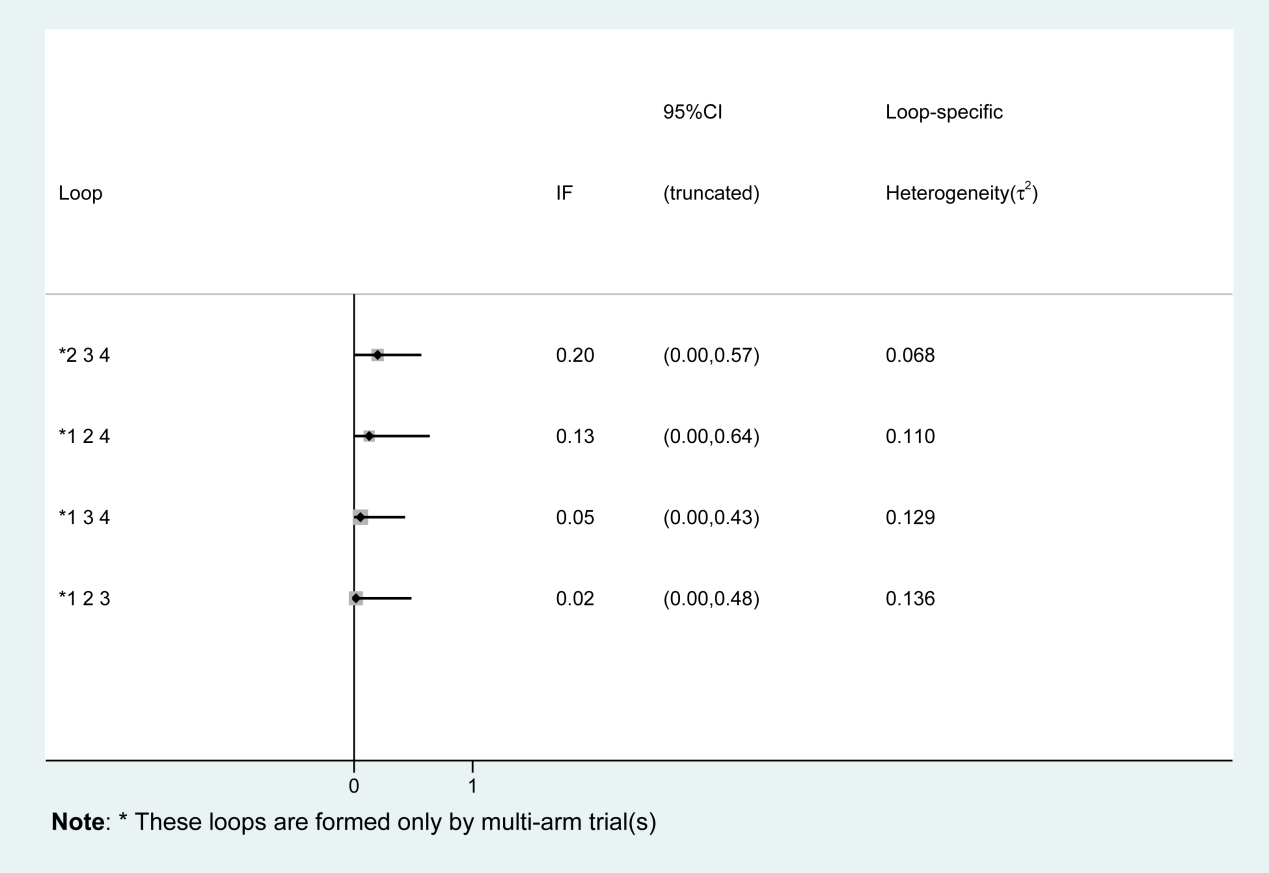
**

**WOMAC physical function subscale**

**
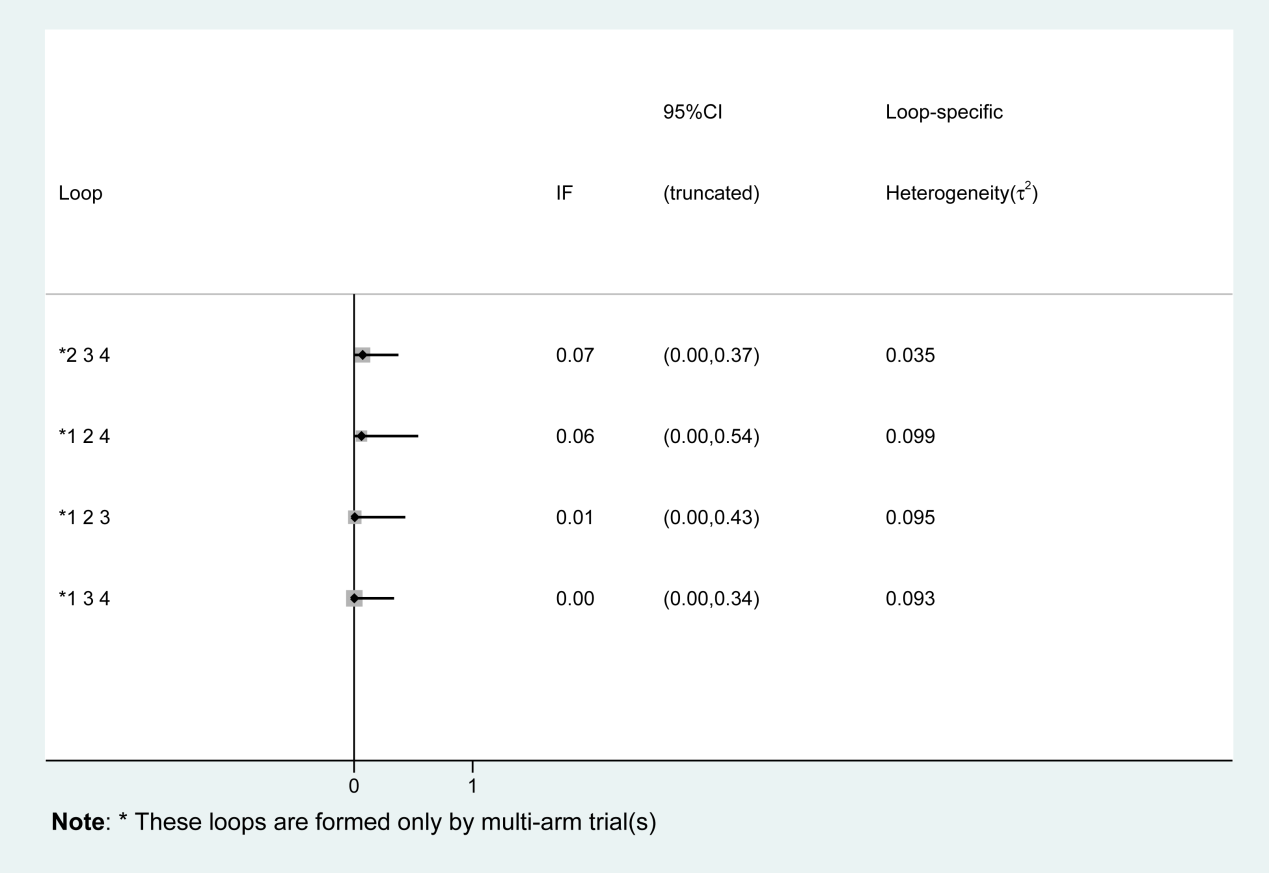
**

**Patient's Global Assessment of OA**

**
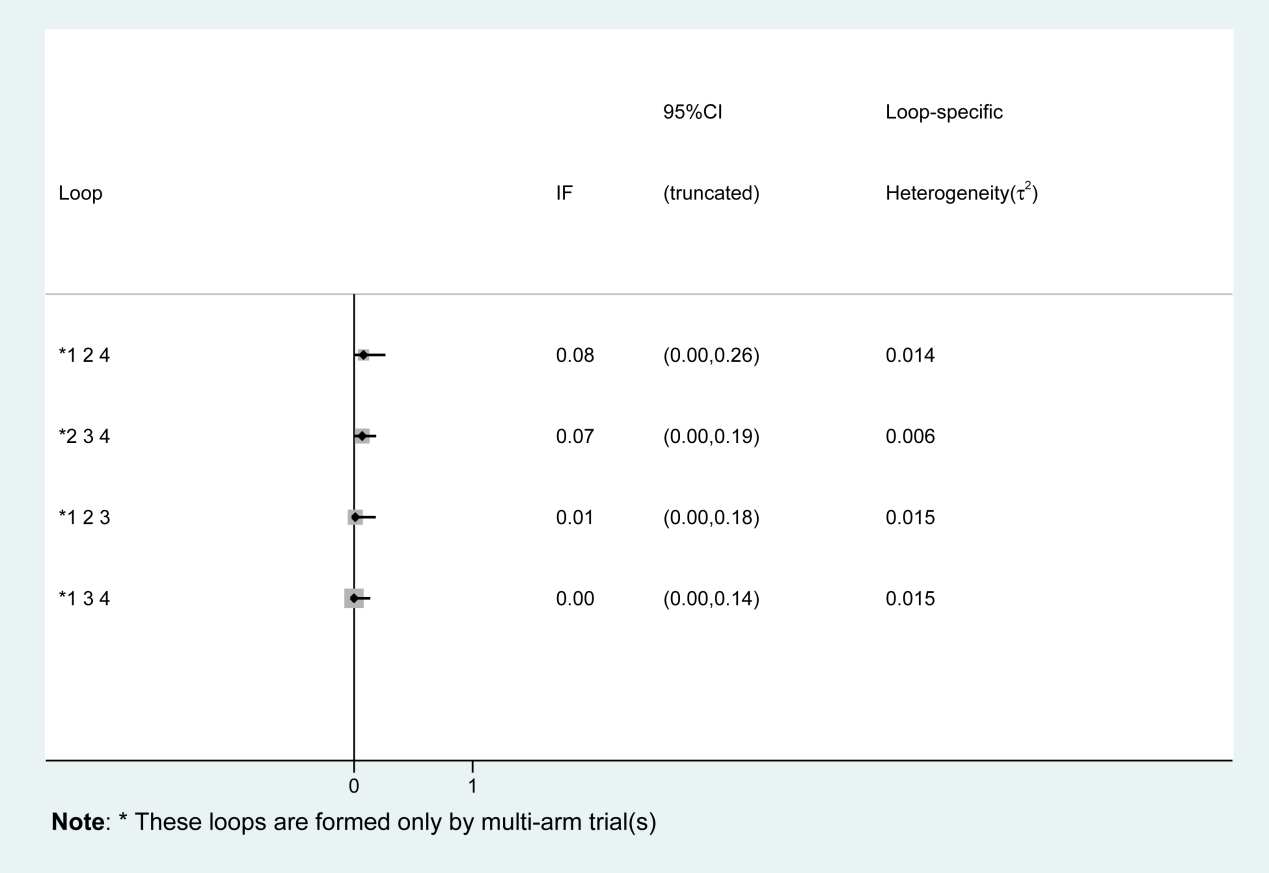
**

**WOMAC stiffness subscale**

**
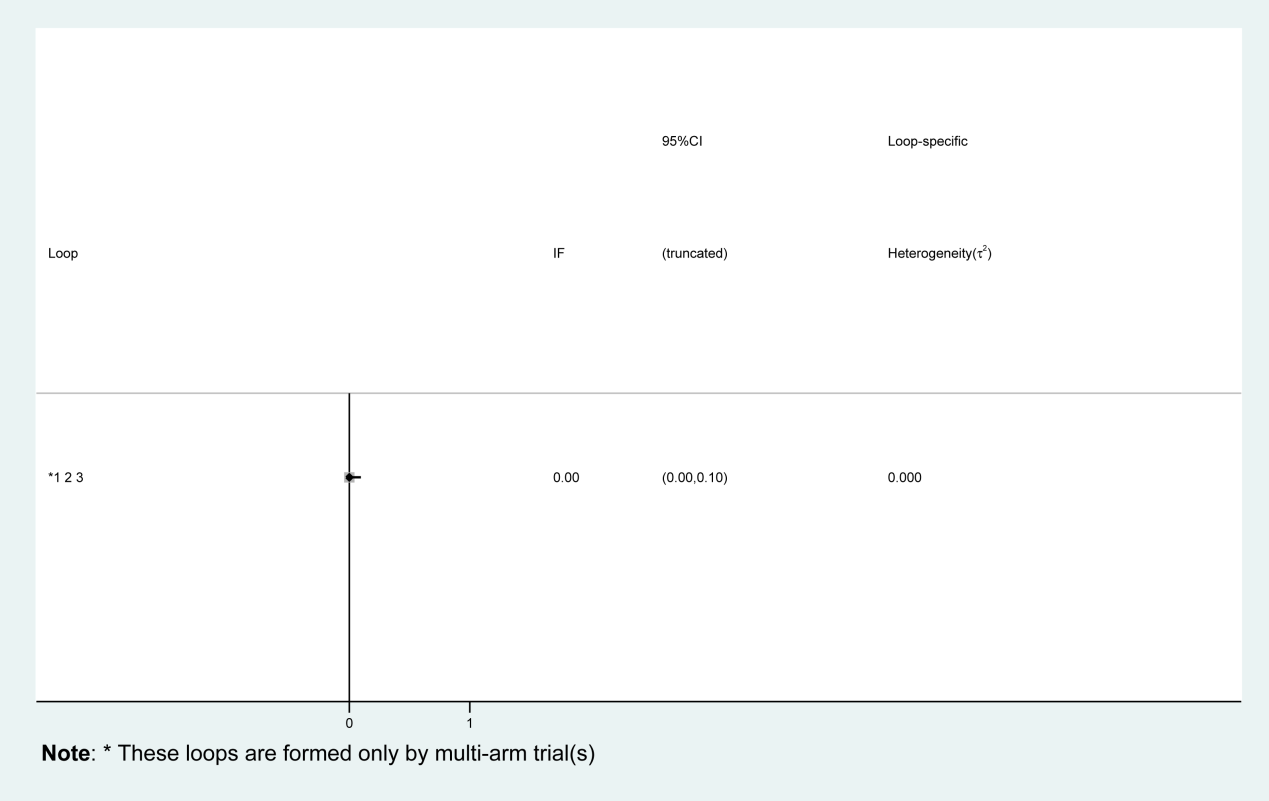
**

**WOMAC pain reduction ≥30%**

**
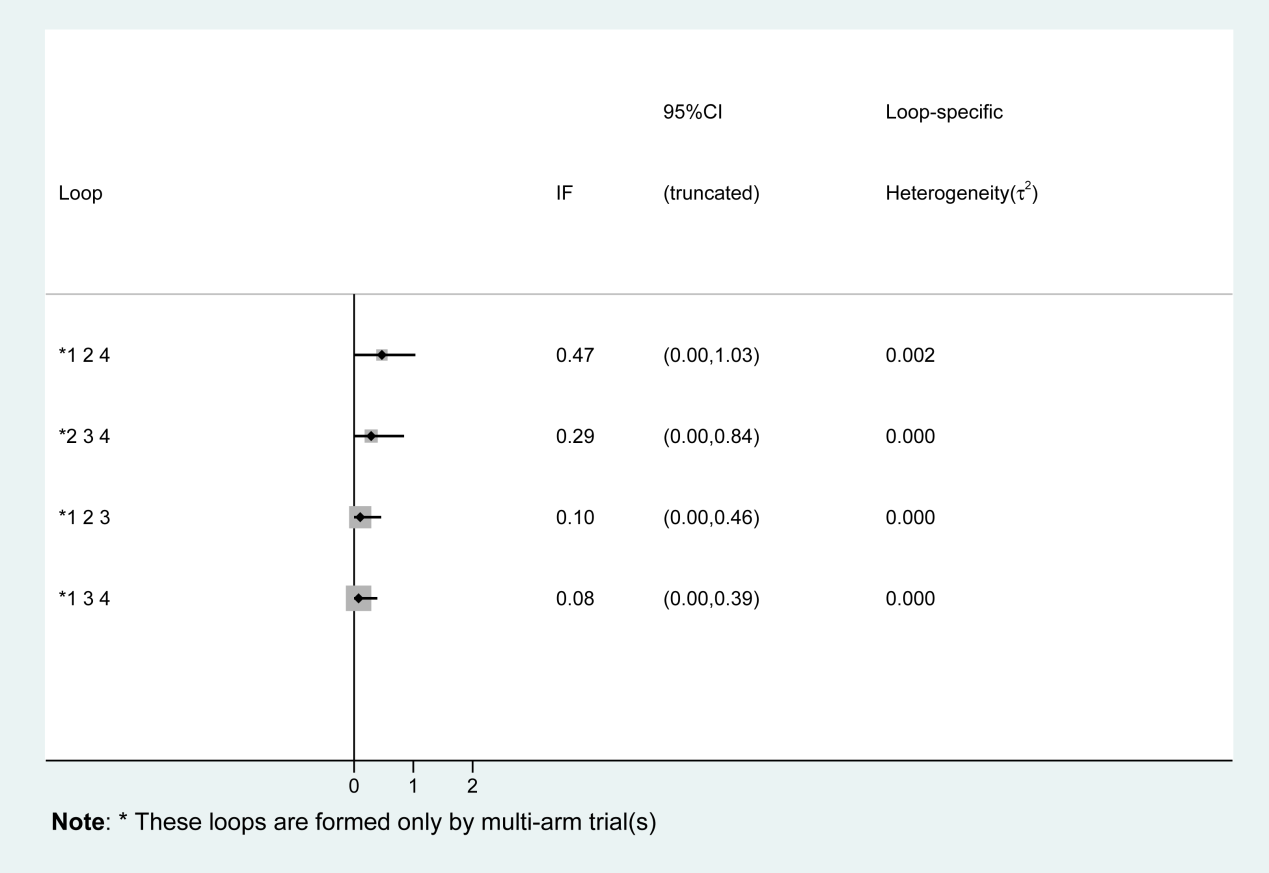
**

**WOMAC pain reduction ≥50%**

**
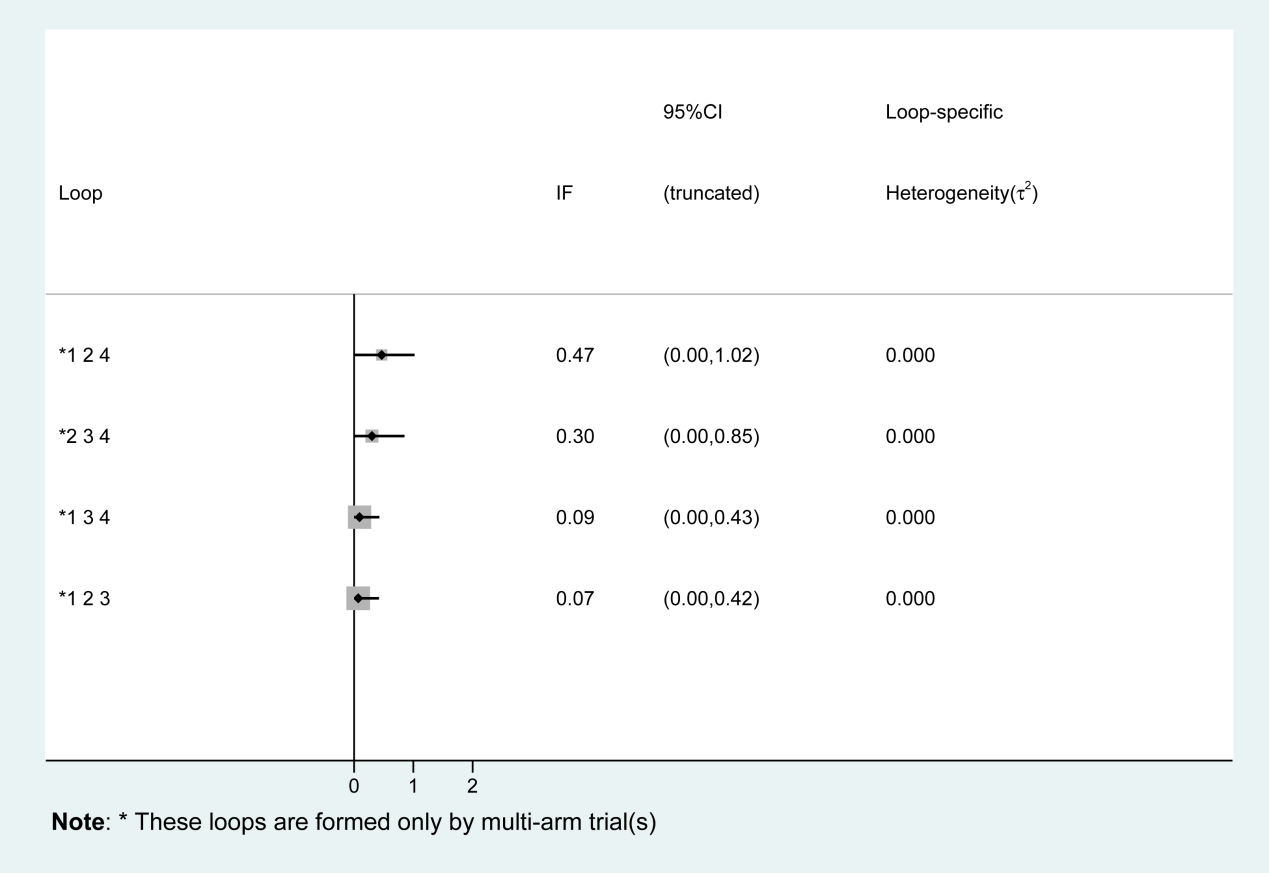
**

**WOMAC pain reduction ≥70%**

**
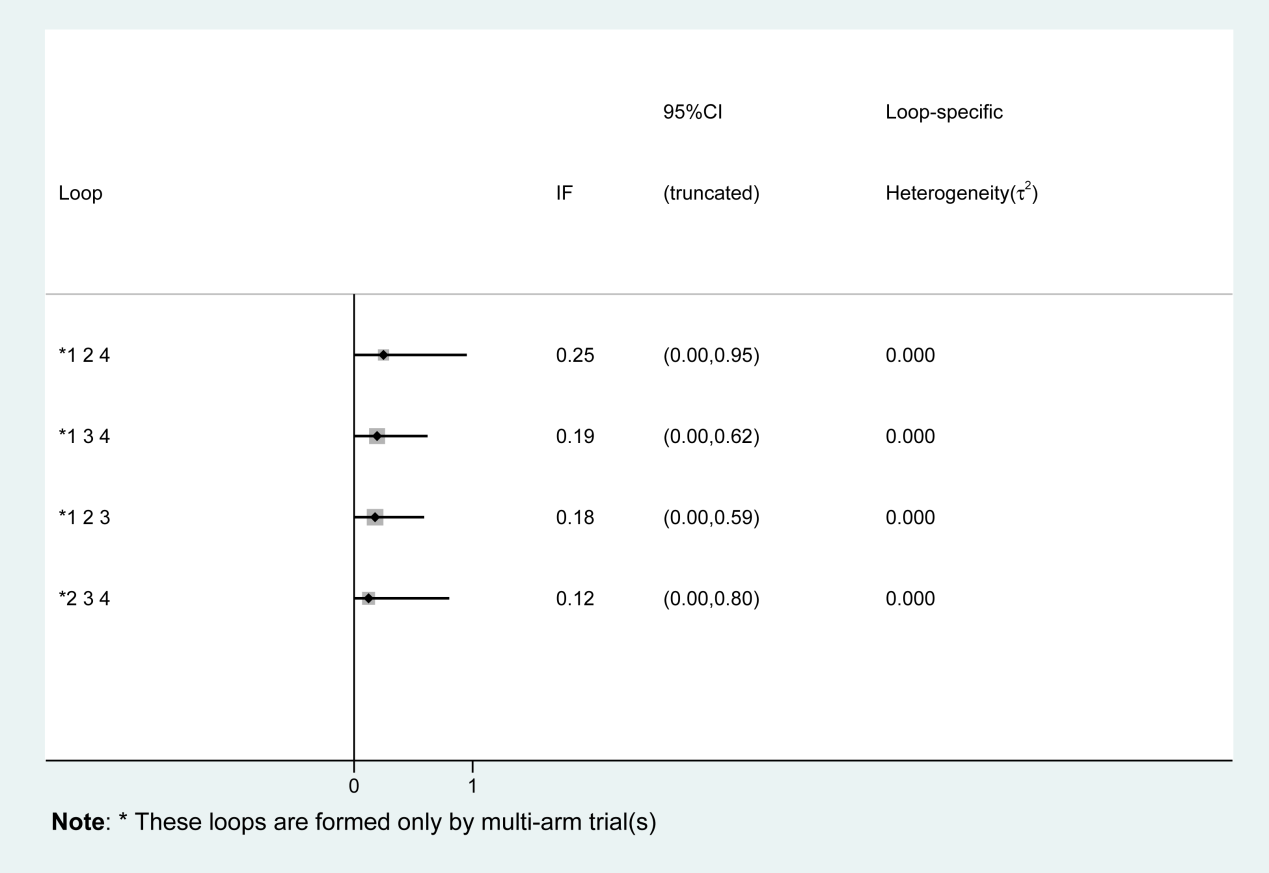
**

**WOMAC pain reduction ≥90%**

**
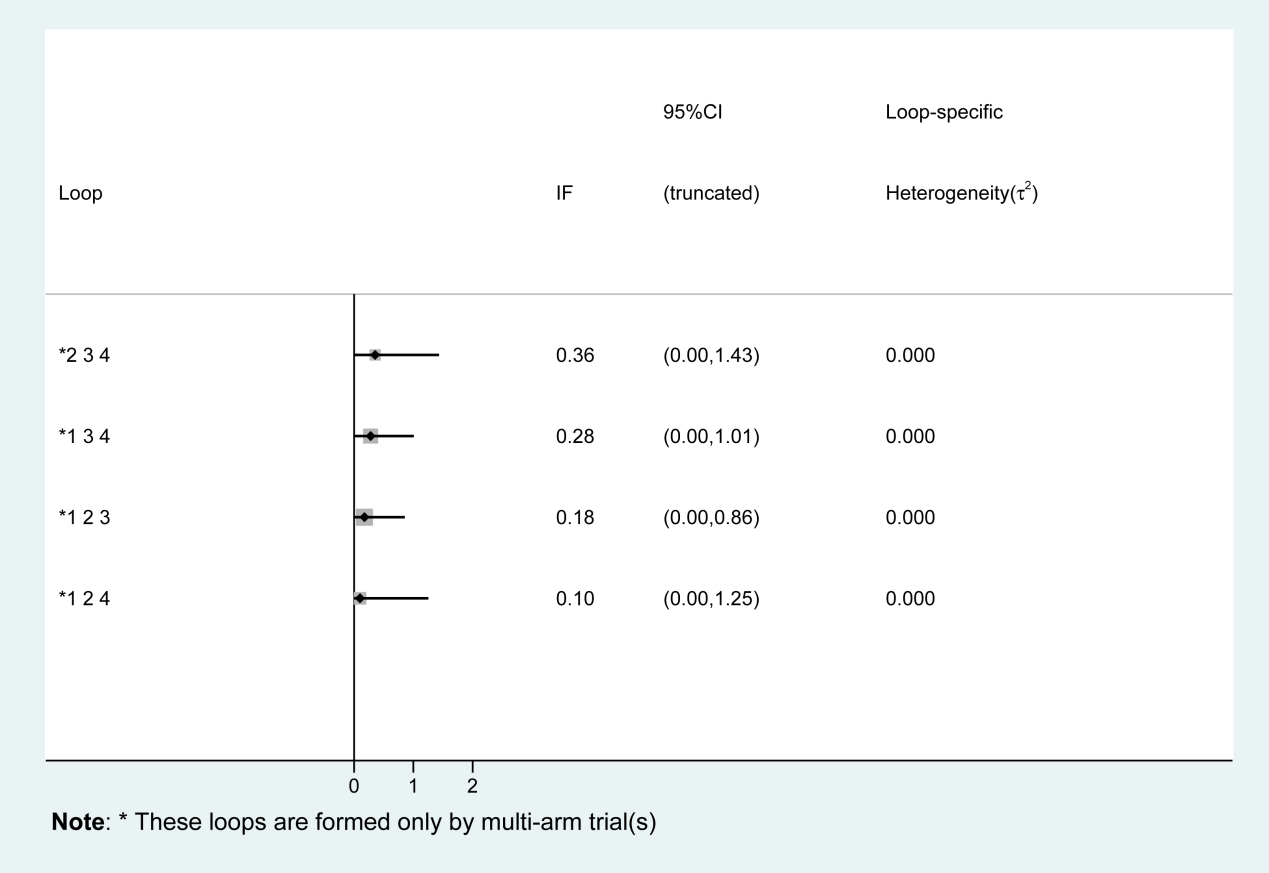
**

**Joints replaced**

**
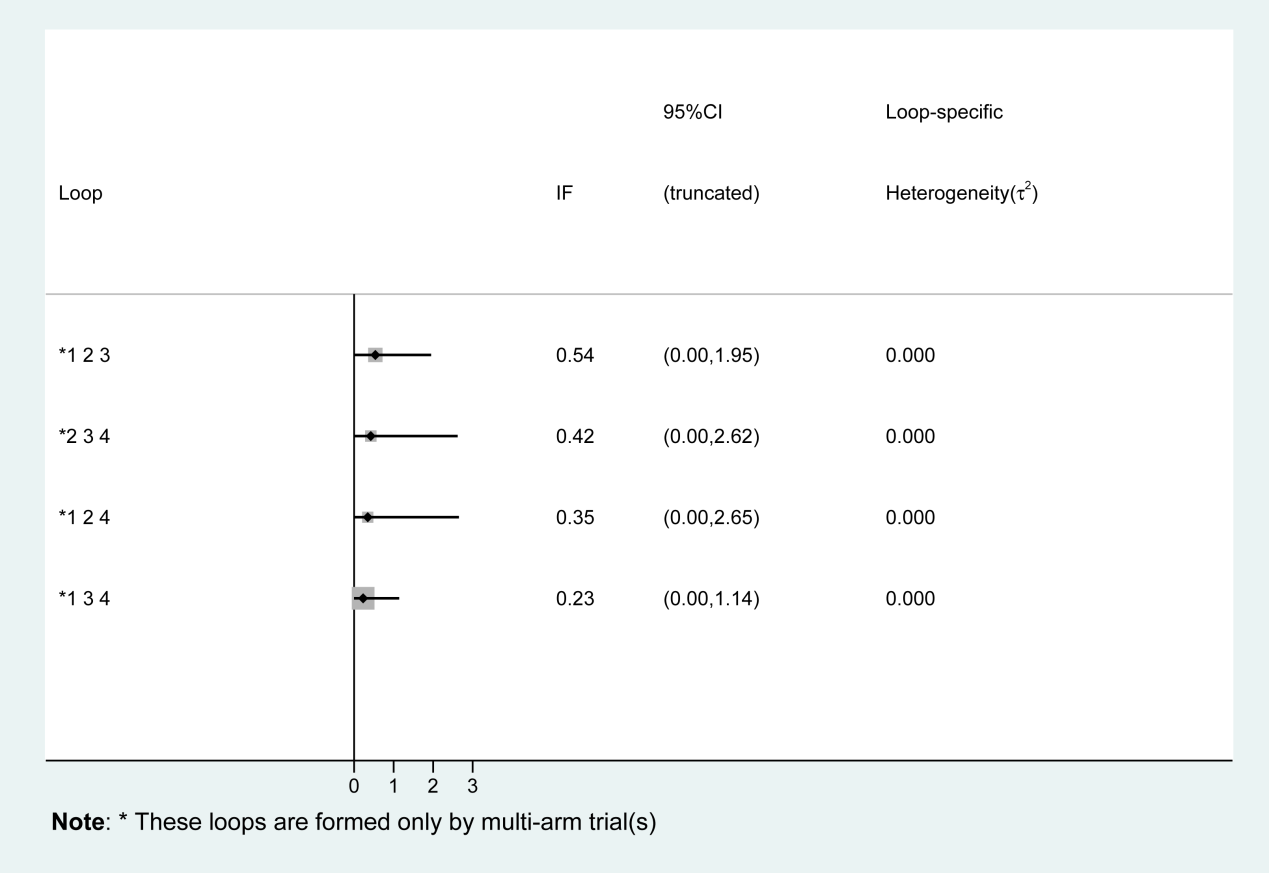
**

**Supplementary Figure 2** The results of loop consistency for all safety outcomes

**Adverse events**

**
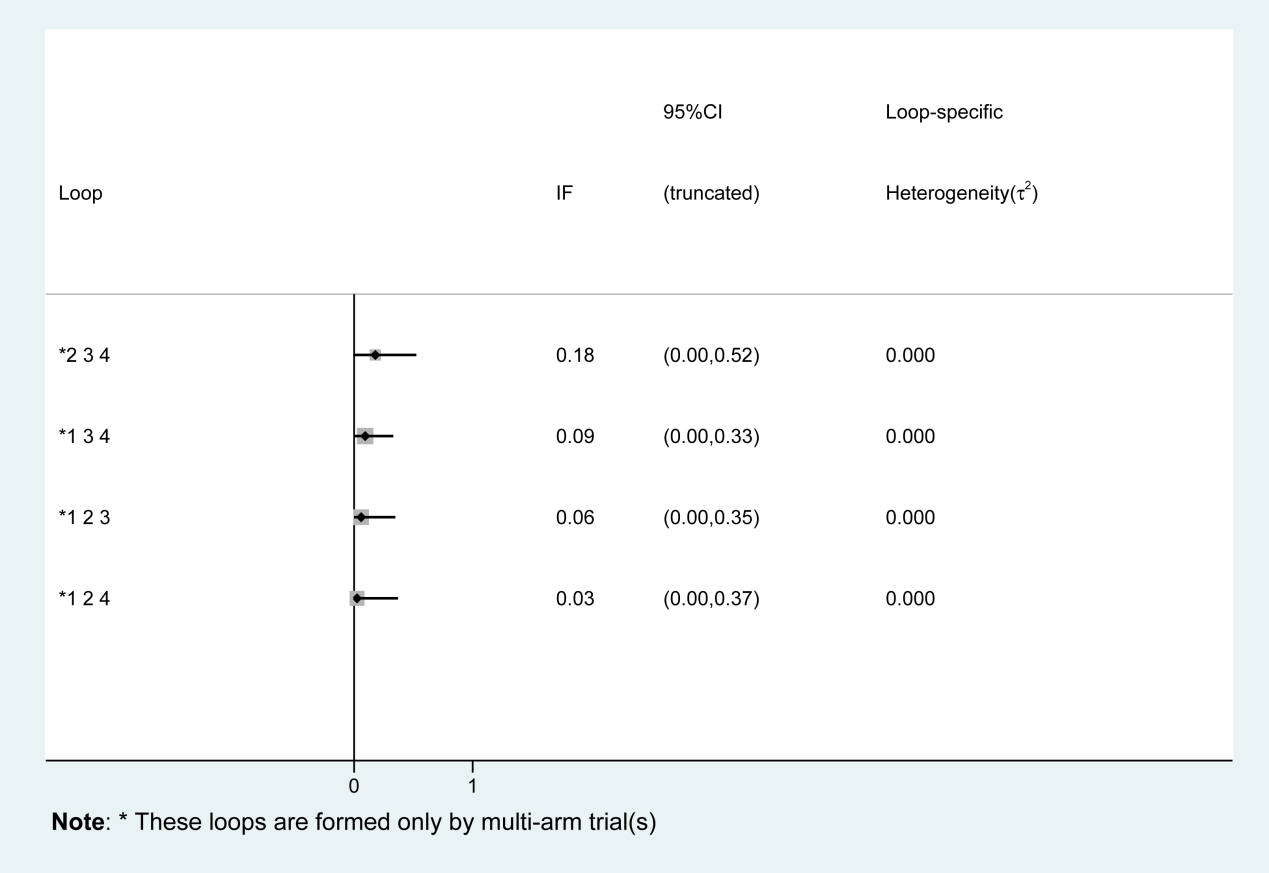
**

**Treatment-related adverse events**

**
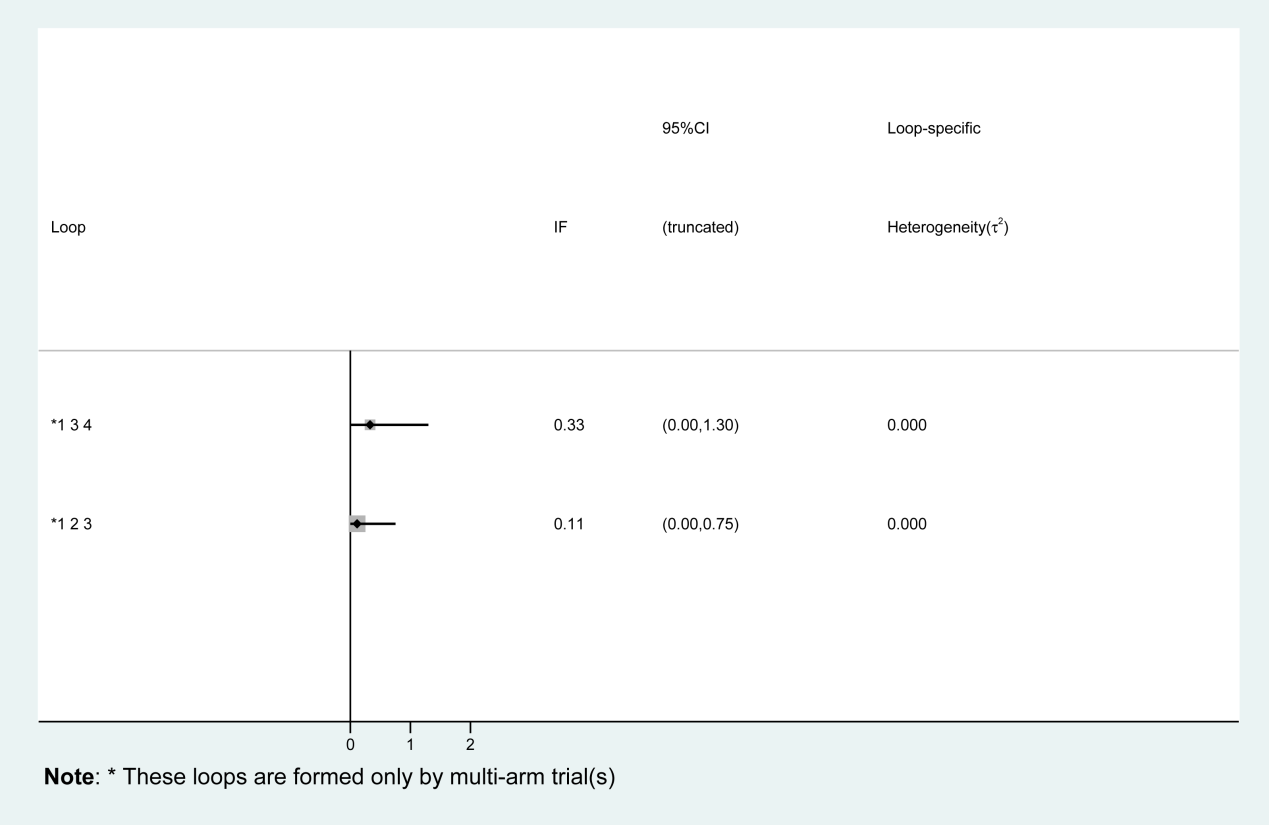
**

**Serious adverse events**

**
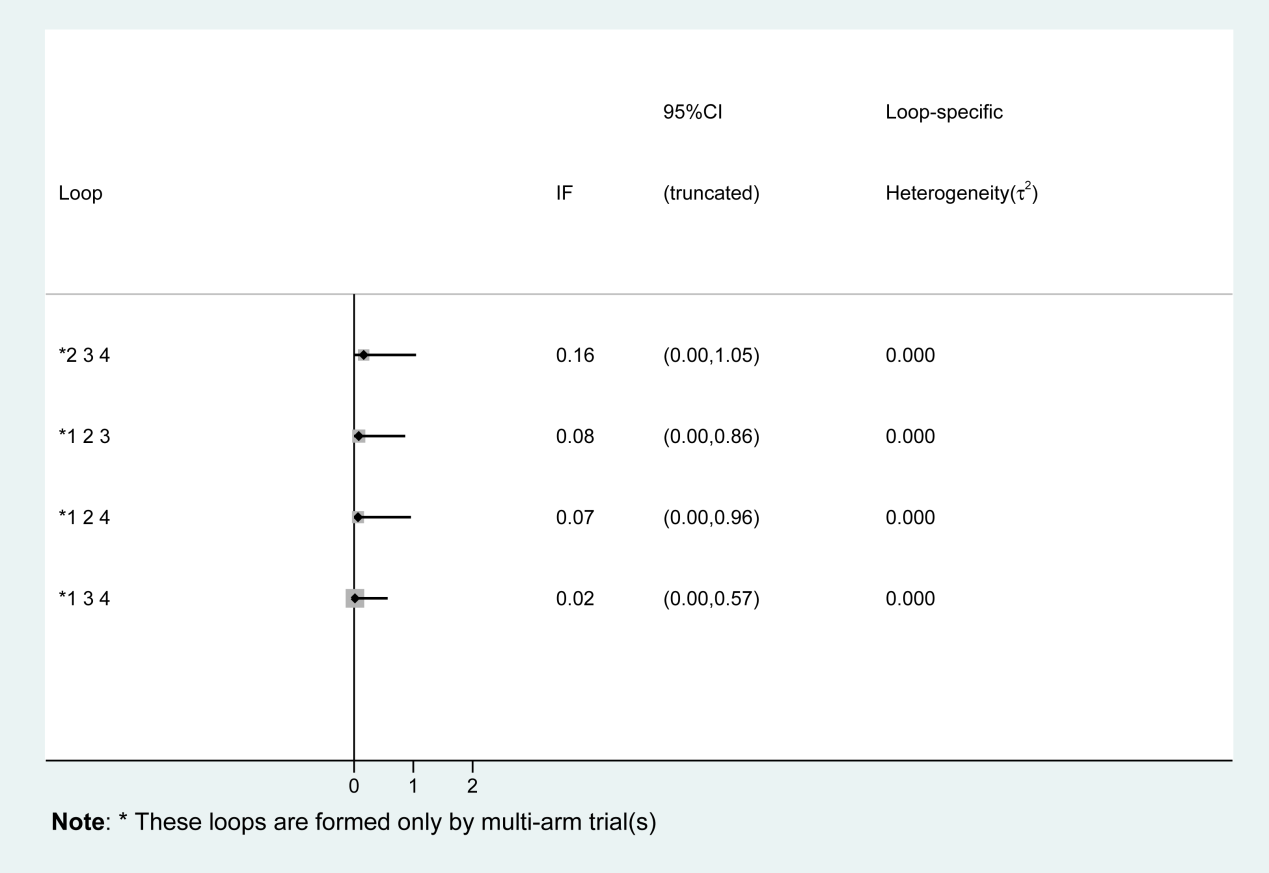
**

**Discontinued due to adverse events**

**
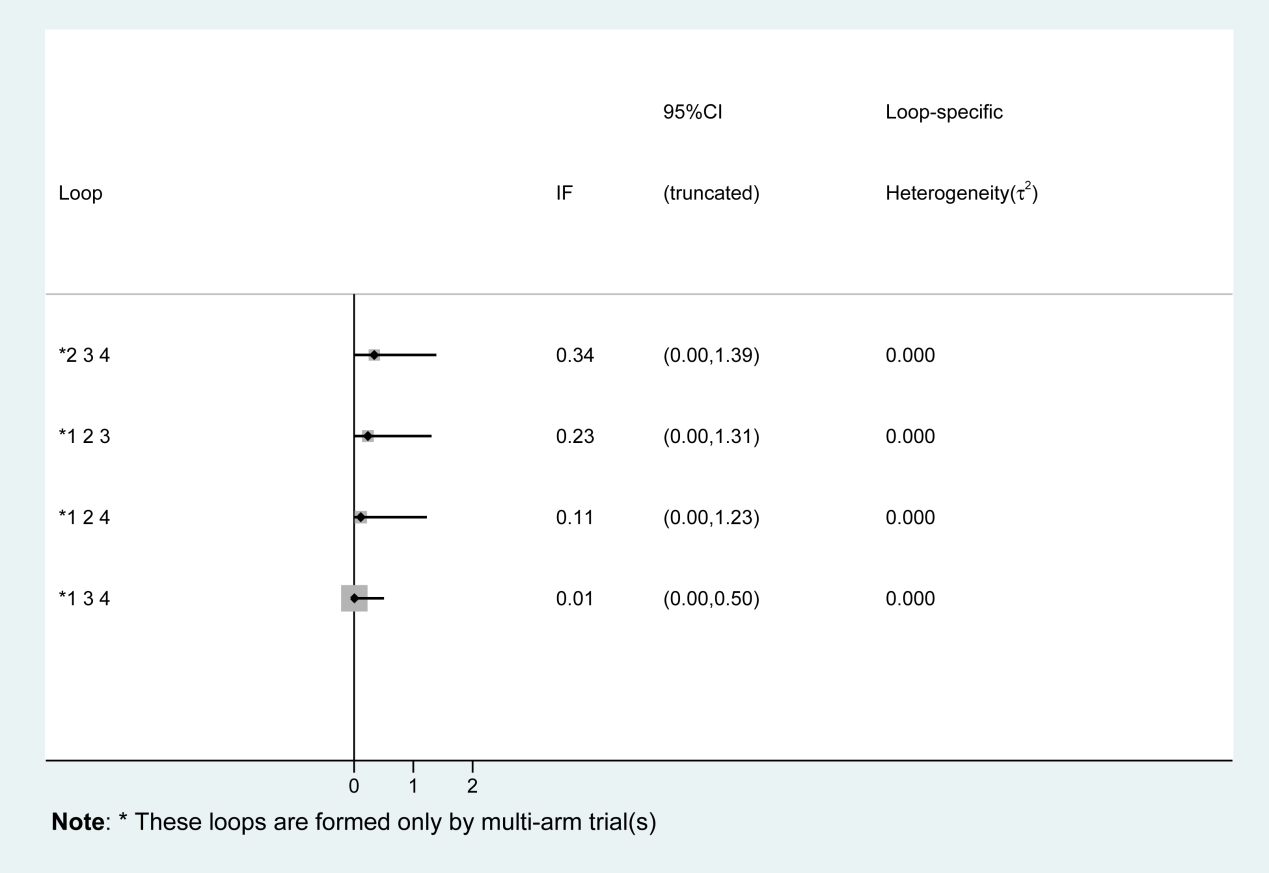
**

**Adverse events of abnormal peripheral sensation**

**
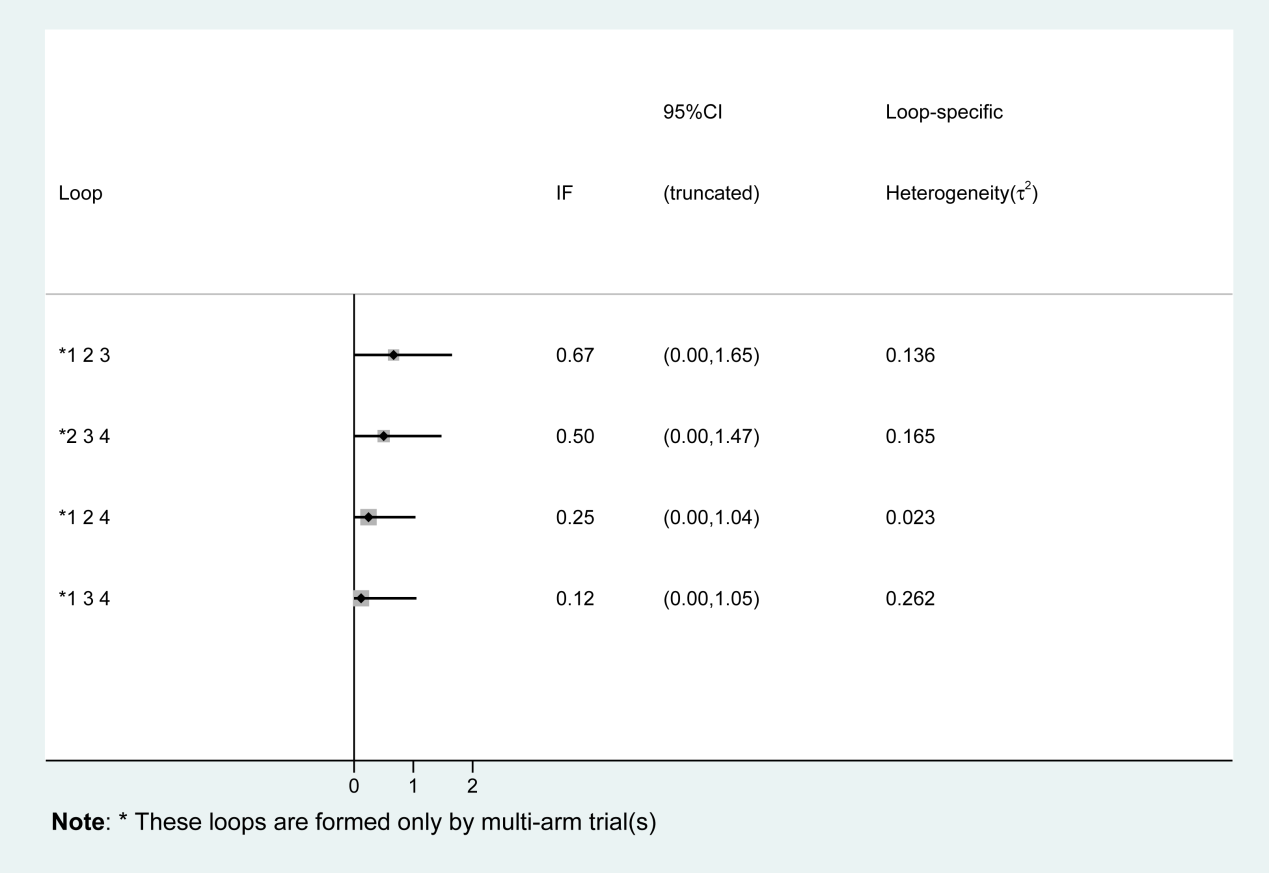
**

**New or worsened abnormalities**


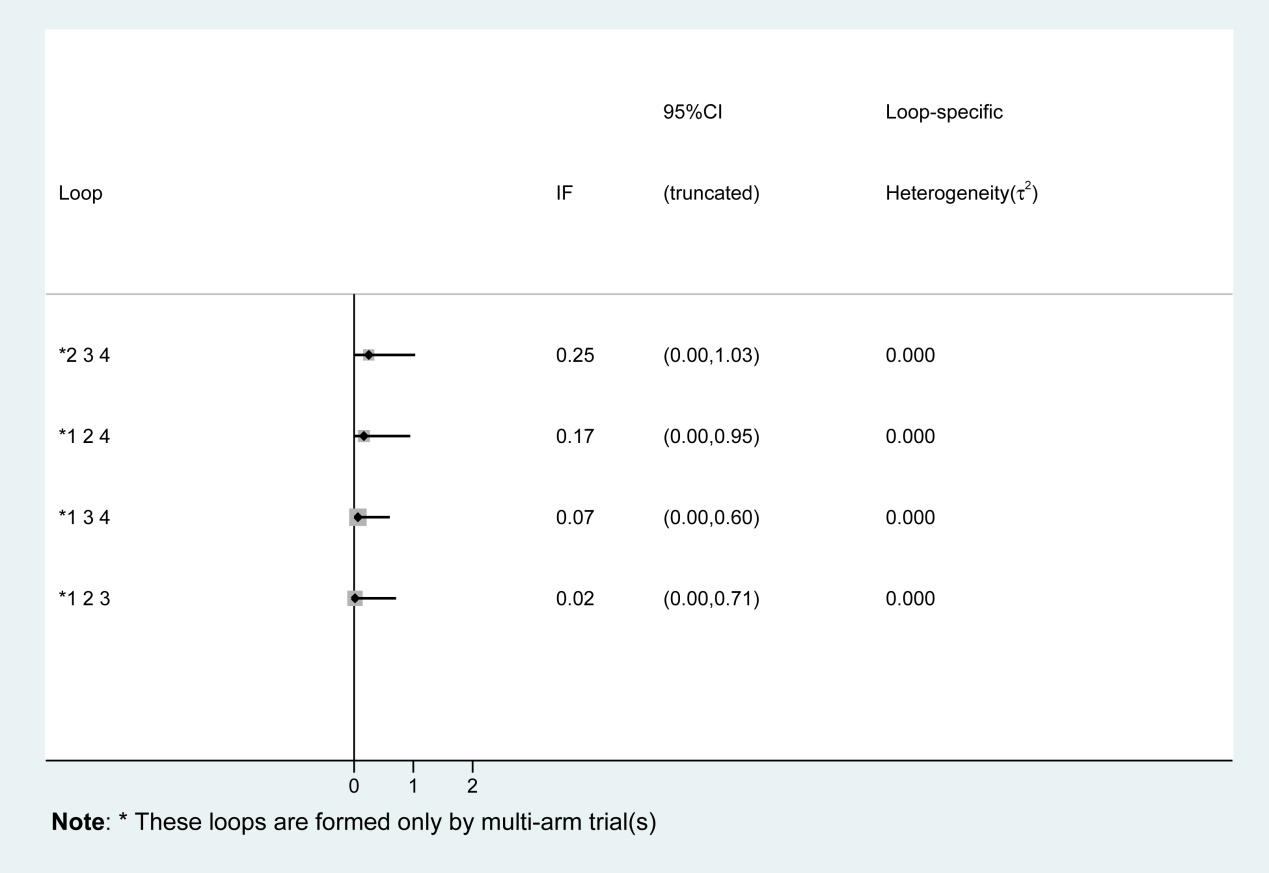

Supplement: Supplementary file 1 [file DataSheet1.docx]
